# First Peoples’ knowledge leads scientists to reveal ‘fairy circles’ and termite *linyji* are linked in Australia

**DOI:** 10.1038/s41559-023-01994-1

**Published:** 2023-04-03

**Authors:** Fiona Walsh, Gladys Karimarra Bidu, Ngamaru Karimarra Bidu, Theodore A. Evans, Thelma Milangka Judson, Peter Kendrick, Alice Nampijinpa Michaels, Danae Moore, Matilda Nelson, Carolyn Oldham, Josef Schofield, Ashley Sparrow, Muuki Karimarra Taylor, Desmond Purungu Taylor, Lee Nangala Wayne, Carol Milangka Williams, Martu elders and experts, Wokka Taylor, Karnu Taylor, Nola Taylor, Wirnta Williams, Muni Rita Simpson, Mayapi Robinson, Junju Judson, Dawn Oates, Jakayu Biljabu, Daphne Biljabu, Patricia Peterson, Nayapi Robinson, Kirriwirri Mac Gardener, Titikiya Edwards, Rosie Williams, Rena Rogers, Dulcie Gibbs, Nancy Chapman, Rosie Nyaju, Jeffery Jangala James

**Affiliations:** 1 Fiona Walsh Ecology, Alice Springs, Northern Territory Australia; 2 School of Engineering, The University of Western Australia, Crawley, Western Australia Australia; 3 Martumili Artists, Newman, Western Australia Australia; 4 School of Biological Sciences, The University of Western Australia, Crawley, Western Australia Australia; 5 Biota Environmental Sciences, Leederville, Western Australia Australia; 6 Australian Wildlife Conservancy, Alice Springs, Northern Territory Australia; 7 Arthur Rylah Institute, Department of Land, Water, Environment and Planning, Heidelberg, Victoria Australia

**Keywords:** Ethics, Psychology and behaviour, Grassland ecology, Entomology

## Abstract

In the past, when scientists encountered and studied ‘new’ environmental phenomena, they rarely considered the existing knowledge of First Peoples (also known as Indigenous or Aboriginal people). The scientific debate over the regularly spaced bare patches (so-called fairy circles) in arid grasslands of Australian deserts is a case in point. Previous researchers used remote sensing, numerical modelling, aerial images and field observations to propose that fairy circles arise from plant self-organization. Here we present Australian Aboriginal art and narratives, and soil excavation data, that suggest these regularly spaced, bare and hard circles in grasslands are pavement nests occupied by *Drepanoterme*s harvester termites. These circles, called *linyji* (Manyjilyjarra language) or *mingkirri* (Warlpiri language), have been used by Aboriginal people in their food economies and for other domestic and sacred purposes across generations. Knowledge of the *linyji* has been encoded in demonstration and oral transmission, ritual art and ceremony and other media. While the exact origins of the bare circles are unclear, being buried in deep time and *Jukurrpa*, termites need to be incorporated as key players in a larger system of interactions between soil, water and grass. Ecologically transformative feedbacks across millennia of land use and manipulation by Aboriginal people must be accounted for. We argue that the co-production of knowledge can both improve the care and management of those systems and support intergenerational learning within and across diverse cultures.

## Abstract (web summary)

Integrating Australian Aboriginal art and narratives with soil excavation data suggests that the regularly spaced bare circles in Australian arid grasslands (sometimes known as ‘fairy circles’) are in fact *linyji* or *mingkirri*, termite pavement nests used by Aboriginal people for domestic and sacred purposes over generations.

## Main

First Peoples have experienced their local environments over millennia (for definitions, see Table 1). Their biocultural knowledge systems^1,2^ are complex and support local demands for food, medicine, clothing and shelter^3^. Australian Indigenous people have lived continuously on the continent for at least 65,000 years^4^, whereas non-Indigenous naturalists and scientists have studied Australia for less than 300 years. Scientists can aspire to make important discoveries of novel phenomena, following Humboldt, Wallace and Darwin. Many environmental ‘discoveries’ are explained according to the traditions of scientists’ own society, culture and knowledge. They generally ignore or even displace the practices and knowledge of First Peoples^5,6^.

**Table 1:** Definition of terms. Italics indicate First Nations languages

chaff	Dry grass, often spinifex, cut by harvester termites to uniform lengths then transported into chambers.
chambers	Expanded spaces with thick plastering, used as living spaces, for young, rest, storage (food or frass), etc. Typically clustered closely together as a nest, but some termites (including some *Drepanotermes* species) build more dispersed chambers.
consolidated soils	Dense soils, also called cemented or concreted soils, of a termite pavement.
desert	Australian scientists define deserts ‘low rainfall, lack of moisture and high evaporation rates. Their dominant ecosystems range from ultra-arid deserts to grasslands and open woodlands [the latter two] cover much of Australia. … Drylands in Australia span arid and semi-arid regions’^9^ (page 12). Australia does not have many hyper-arid regions as found in Namibia. Generally, Aboriginal people do not name their countries as desert, as indicated by the book title ‘You Call It Desert: We Used to Live There’^63^.
‘fairy circles’	Used by scientists and others to describe regularly distributed bare circular or elliptical spots in arid grasslands attributed to biotic interactions. Tinley (1971) was the first scientist to write about the bare patches in Namibia. The term was then used in a scientific paper in 1994, but its definition and attributed causes remain contested^32^.
First Peoples, Indigenous people, Aboriginal people, [Language] people	These terms span from international to regional scales. First Peoples indicate international contexts, Indigenous people indicates Australian Aboriginal and Torres Strait Island people, and Aboriginal people indicates Australian desert people. Additionally, we name groups by their geography and languages (for example, Fig. 3b). These terms might be compared, for example, to the use of British, Scottish and Glaswegian to describe societies at finer spatial scales.
flying termites	This term is more consistent with Aboriginal English of ‘flying ants’ applied to termites. ‘Alates’ is the entomological term for the reproductive caste of termites that muster within pavements or mounds and emerge with wings to fly. They are rich in fat and were highly prized foods for people and animals. *Pilarrpa* and *Watanuma* are two of several language terms for flying harvester termites.
foraging tunnels	Temporary, narrow passageways with little or no termite-built reinforcement or ‘plastering’, used for foraging at the soil surface and under grass leaves, extending from permanent galleries to food sources.
galleries	Permanent, narrow passageways with termite-built reinforcement used to connect chambers.
*Jukurrpa* also known as Dreaming	The word ‘Dreaming’ is controversial^89^ and has been resisted by some desert Aboriginal groups who say it diminishes their way of thinking as childlike. *Jukurrpa* is a Martu term with synonyms in other desert languages. Simplistically, it translates to ‘law from the Dreamtime’^90^ (page 72) with many other meanings related to narratives, sites, conception beings, identity and more including ‘The Dreaming conjures up the notion of a sacred, heroic time of the indefinitely remote past, such a time is also, in a sense, still part of the present. One cannot ‘fix’ The Dreaming in time: it was, and is, everywhen.’^91^. ‘A period beyond living memory in which ancestral beings were responsible for the genesis of the spiritual, physical and moral world, and for providing the laws and ceremonies which sustain contemporary existence.’^92^.
*linyji*	A Manyjilyjarra language term or *linytji* in Pintupi. They describe these as bare, very hard circular areas. *Linyji* is also applied to claypans that are also hard, bare clay-based areas that hold water after rainfall. For the title, we use the Martu term. Different languages have different terms for the same feature (for example, *putu* in Pitjantjatjara, *mingkirri* or *parlarnji* in Warlpiri), but here we use *linyji* only with Martu authors and primary contributors.
*manyjurr*	Manjilyjarra or Martu language term for termites who occupy *linyji*; *manyjurr* are colloquially called ‘white ants’.
Martu people	Martu are the traditional owners of a vast area of central Western Australia. ‘Across this country, Martu share a common law, culture and language. The Martu were some of the last of Australia’s Indigenous people to contact European Australians. Martu speak or understand numerous languages. For most Martu, even the children, English is a second or more language. Martu languages include: Manyjilyjarra, Warnman, Kartujarra Putijarra, Nyangumarta. In 2002, Martu native title rights over 13.6 million hectares of the Western Desert were recognized. This is referred to as the Martu native title determination area.’^93^. The western neighbours of Martu include Nyiyaparli and Nyamal people (Fig. 3b).
*mingkirri*	Warlpiri term for [white] ant-bed, termite mound; synonym is *parlarnji*.
Native Title Determination Area	A decision by an Australian court or other recognized body that native title existed under the Native Title Act^94^.
pavements	This word is used instead of ‘fairy circles’. It is an English translation of *linyji (*syn. *mingkirri)*. Pavements are the hard capping of subterranean termitaria (nests) of harvester termites (*Drepanotermes* species). Other English synonyms include termitaria or ‘slabs’ or mounds (even if no above-ground mound is present).
threshing	A stage of seed food processing wherein the seed heads are hit or threshed against a hard flat surface to separate edible seed from inedible plant parts. In Indigenous Australia, this was done manually be people. In other continents, threshing was done with flays, stock or machinery. Synonym: husking.

Australia is often characterized as a land of extremes, in part because much of Australia is arid and driven by strong climate and disturbance cycles^7^. Australian desert systems have often been viewed as ‘unpredictable’^7^ because modern ecological studies rarely have the longevity to see long-term patterns in ancient, slow, ecosystem and landscape processes punctuated by production pulses after rainfall events^8,9^. Australian Indigenous people have lived in inland deserts for at least 50,000 years^10^. Their knowledge of desert ecosystem function has evolved and been transmitted over more than 2,000 generations of people. Experiences and observations, trials and discursive analysis, specialized vocabularies and narratives, ritualized body and ground paintings, sculptures and rock art, dances and songs help encode accumulated ecological understandings at multiple spatial and temporal scales^11–14^.

With some exceptions^15–17^, earlier Australian arid zone scientists rarely drew upon ecological information encoded in Aboriginal knowledge. But such approaches are shifting in Australia as Indigenous people and their knowledge are increasingly being recognized throughout ecosystem science and management^18,19^. Nationally and internationally, there are expanding ethical, policy and/or legislated requirements at most levels of government, including the Intergovernmental Platform on Biodiversity and Ecosystems Services, to incorporate First People’s knowledge in applied ecology, ecological research, ecosystem assessments and monitoring^20–22^.

This engagement with Indigenous people and their knowledge spans evolving approaches that include knowledge extraction to benefit science, two-way knowledge sharing, collaborative research and equitable attribution and benefit sharing, knowledge co-production and/or Indigenous people leading research^23^. Examples specific to collaborative ecosystem research with Australian desert language groups have been described^24–27^ albeit with varying power relations amongst participants.

Despite inclusive or collaborative trends, some arid-zone ecological studies continue either without Aboriginal contributors or without approaches derived from Aboriginal people’s knowledge. Such omissions may affect our understanding of arid-zone phenomena^28^. Published scientific explanations can persist and dominate scientific analysis^29^, even if incorrect or insufficiently substantiated. Concurrently, Indigenous knowledge fades under colonial dispossession and modern pressures^30^.

**Fig. 1:**
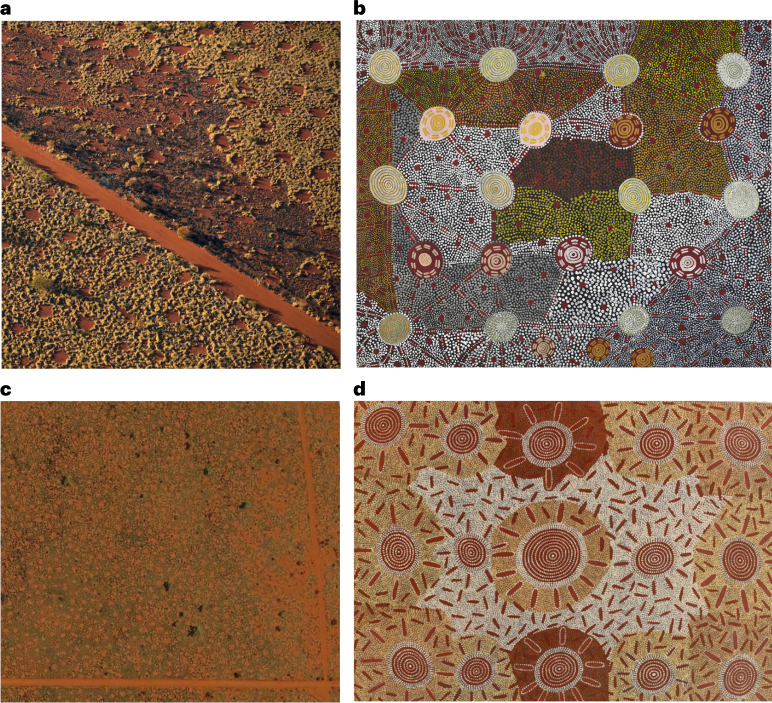
Parallels in depiction of termite pavements in helicopter and satellite imagery and Aboriginal painting. **a**, Aerial photographs show regularly spaced termite pavements in spinifex grassland of *Triodia basedowii* on Nyiyaparli lands. The red vehicle track is same as in **c**. Dark ash of recently burnt spinifex is visible by the track (photo © Mike Gillam 2021). **b**, Painting of regularly spaced termite pavements by Anmatyerre, Warlpiri and Arrernte man Kaapa Tjampitjinpa, titled ‘*Watanuma*’ (edible flying termites). Synthetic polymer paint on canvas, 202.4 × 171.8 cm. (1976), National Museum of Australia, © estate of the artist, licensed by Papunya Tula Artists and Aboriginal Artists Agency^95^. **c**, The pavement spot patterns are visible using Google Earth; the track on the right (east) is the same track as shown in **a** near plot FC 2 (Image © 2/2022 CNES/Airbus, eye alt 610 m). **d**, Painting ‘Flying termite dreaming’ by Anmatyerre Warlpiri man, Paddy Carroll Tjungurrayi associated with the pupate stage of the flying ant [termite] *Watanuma*. The artist indicated the creamy background represents spinifex in which the insect flourishes. Synthetic polymer paint on canvas, 180 × 120 cm, (1980), unknown collection, © estate of the artist, licensed by Papunya Tula Artists and Aboriginal Artists Agency. There are visual and content similarities between photographs (**a**,**c**) and artwork (**b**,**d**). Further interpretations of **b** and **d** are provided in Supplementary Fig. 1. Images **a**, **b** and **d** in this figure are covered by Creative Commons license CC BY-NC-ND.

One example of this has been the ecological debate relating to the regular spacing of bare circular patches in spinifex grasslands of arid Australia (Fig. 1a,c), that have been named ‘fairy circles’. These were originally researched in south-western Africa^31^; the debates about them were reviewed by Meyer et al.^32^. Some scientists propose that their regular size and spatial patterns result from self-organizing hydrological and vegetation control feedback loops. This explanation was extrapolated from hyper-arid and arid south-western Africa to arid Western Australia^33–37^.

Aboriginal people have a different explanation for the bare circular patches in spinifex grasslands of arid Australia. They have observed strong connections between the regularly spaced bare circles and termites. These connections are evidenced by oral statements, frequent depiction in sacred artworks (for example, Fig. 1b,d and Supplementary Figs. 1 and 2) and other descriptions of the circles (Ethnographic data; 10.26182/k3p0-hf57). This evidence raises questions about the applicability to arid Australia of the hypothesis proposed for African systems. Additionally, there is conflicting scientific evidence about the relationship between these ‘fairy circles’ and termites in desert landscapes^33,38,39^. Systematic, thorough and deep explorations of Aboriginal knowledge of circles and termites, alongside scientific research, is needed to help understand the phenomenon of ‘fairy circles’ in Australia.

## Termites—delicacy for people, food for animals

Termites form a primary link in desert food webs for people and other vertebrates^40^. Harvester termites eat senescent or dead grasses and are the dominant herbivores in spinifex grassland ecosystems^7^. Termite biomass per unit area may be comparable to domesticated livestock^41,42^. Many vertebrates in spinifex grasslands rely on termites as food; termites in deserts are analogous to krill in oceans. Most grass harvester termite species have underground nests, but some build nests above ground (mounds) or level with the ground (pavements); their nest structure varies with local conditions (for termite ecology review, see Supplementary Information).

Given Aboriginal knowledge and the superabundance of termites, the roles of termites in Aboriginal people’s lives deserves systematic study. Internationally, many Indigenous people see termites as beneficial^43,44^, while most non-Indigenous people consider termites to be pests^45,46^. The biocultural roles of termites in desert Australia can be difficult to discern from published materials. Records are scattered and fragmented. Termites have generally been overlooked or misnamed by ethnographers writing about Australian Indigenous cultures (for example, art documentation calling them ‘flying ants’). Despite accounts that include cultural uses of termites in semi-arid regions^46–48^ international reviews of edible and medicinal termite use by First Peoples have omitted Australian Indigenous uses^43^.

Our evidence from Martu and Warlpiri ancestors and in art and ethnographic records from Aboriginal groups shows that harvester termites have been, and continue to be, profoundly important to desert people. In Australia, other people generally saw termites as a costly insect whereas desert Aboriginal people revered termites^45^. For these people, flying termites and pavements have evoked feelings including awe and wonder, nostalgia and sadness (for example, Ethnographic data, row 33; 10.26182/k3p0-hf57). In arid regions, harvester termite pavements, mounds and termite soils, as well as flying termites and other termite castes, have been intensively used for both secular and sacred purposes. Fifteen desert nations and language groups have vocabularies related to harvester termites (Supplementary Fig. 3). We documented forty-two uses of harvester termites and their pavements by desert Aboriginal people (Ethnographic data; 10.26182/k3p0-hf57). We assume more records are to be found.

In north-western Australia (Supplementary Fig. 3) in the western Great Sandy Desert, where regularly spaced bare circles are found, Martu speakers state that *manyjurr* (termites) lived under the *linyji* (pavements). Martu classified at least four taxa of flying termites including those flying out from *linyji*. Termite alates (Supplementary Fig. 5h) are seasonally abundant, rich in oil and were eaten by Martu and their neighbours who classed *pilarrpa* (flying termites) as gourmet food (*wama*). Termite alates and other castes are eaten by Martu food animals (Ethnographic data, row 28; 10.26182/k3p0-hf57), such as *Minguwa* (Echidna), *Mankarr* (Greater-eared bilby), *Parnajarpa* (Sand goanna) and *Mulyamiji* (Great desert skink). Pre-emergent alates and flying alates were abundant in episodic flushes^45^. Flying termites were collected by the ‘bucketful’ to eat (Ethnographic data, row 26; 10.26182/k3p0-hf57). Pintupi, Warlpiri and other people (locations in Supplementary Fig. 3), say that flying termite abundance corresponds with high rainfall periods and contributes to both food animal and human condition, also known as ‘fat times’^12,49^. In the past, people celebrated these benefits and magnified them through ‘increase ceremonies’^45,50^, which were believed to have led to periods of higher human fecundity^12^. These practices lead us to questions about the variety of pathways by which high rainfall periods and termite production may have increased human reproduction.

**Fig. 2:**
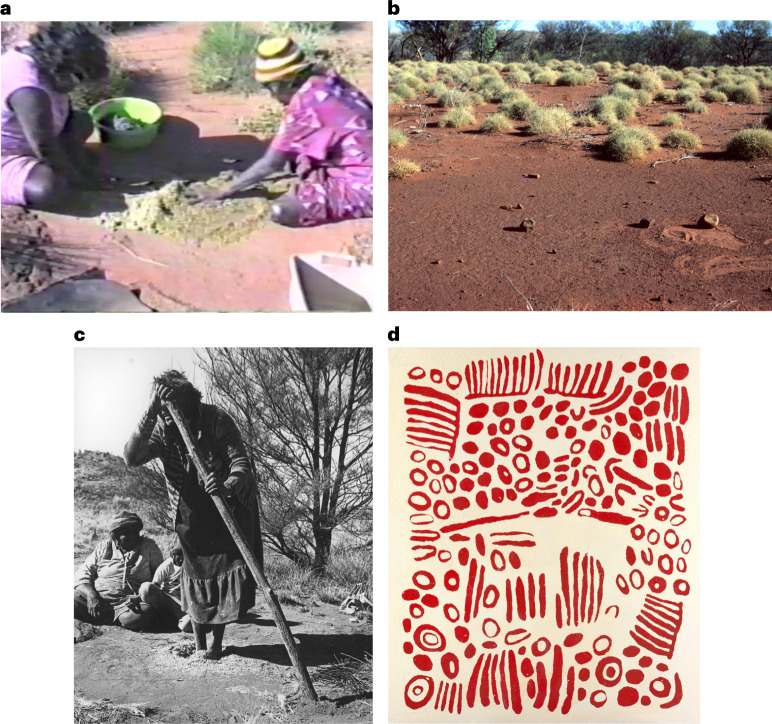
Aboriginal women use pavements to thresh seed using different methods. **a**, Martu co-author Thelma Judson and Wirnta Williams rub Kalpari (*Dysphania kalpari*) seed against the hard *linyji* surface to separate seed from chaff (screenshot from video by J. Walsh, 1988; https://vimeo.com/399035889; photo © Fiona Walsh). **b**, Hand stones left on a *linyji* by Martu ancestors during pre-colonial times. The sand marks are from Martu people in 1987 who demonstrated seed threshing motions for which the stones were used (photo © Fiona Walsh). **c**, Martu elder Nganyinytja Lewis uses feet and pole to thresh seed in a pit^96^ (photo by T. and B. Blake, 1987, © Ara Irititja Archive Nos AI-0081679; see video Putu for Wangunu = Pavement for Seed Food; https://vimeo.com/654072562 and https://vimeo.com/539494391). **d**, The painting titled ‘*Watanuma*’ (edible flying termites) (2008) by Pintupi woman Wintjiya Napaltjarri illustrates different associations with termites and pavements in synthetic polymer paint on canvas, 151.2 × 182.0 cm. National Gallery of Victoria, Melbourne. © estate of the artist, licensed by Papunya Tula Artists and Aboriginal Artists Agency. Further interpretations of **c** and **d** are provided in Supplementary Fig. 2. All images in this figure are covered by Creative Commons license CC BY-NC-ND.

The authoritative Aboriginal knowledge of termites and their ecology stems from the significance of harvester termite alates to people for food and to people’s uses of pavements as surfaces to thresh seed and process other materials. Seeds were a staple food group in pre-colonial desert economies^50,51^. Archaeologists and ethnographers have studied the use of seed-grinding stones and stages of seed food preparation^52^. Our study adds evidence about the vital threshing stage of seed processing, which is rarely described. While some threshing (for definitions, see Table 1) was done on other surfaces (for example, rock slabs), termite pavements were preferred as they were abundant, hard, flat and their surface less likely to shatter and contaminate seeds. Photo and video evidence shows that fertile grasses, herbs and branches of shrubs were threshed by hand on the surface of termite pavements (Fig. 2a,b). Excavated pits in pavements (Fig. 2c) were also used to thresh bulk quantities of seeds (see ref. ^53^ for an early record). Sometimes pivot poles were used for balance within the pit (Fig. 2c). Ethnographic data rows 2–3 (10.26182/k3p0-hf57) note such uses. The multiple use of pavements has been documented in paintings (Fig. 2d) that also encode deeper cultural meanings.

The fundamental roles that pavement mounds and flying termites played, and continue to play, in desert ecosystems and in the lives of Aboriginal peoples highlight the need for a greater understanding of harvester termite ecology.

## Art encodes ecological knowledge

Aboriginal knowledge of harvester termites is embodied in art and narratives, ceremonies and songlines, which help sustain knowledge when species are absent or invisible (underground), for example, during periods of low rainfall^14^. Contemporary Aboriginal art continues to record such ecological knowledge^54^ (Supplementary Fig. 4). We found 73 artworks related to termites painted by 34 desert artists who associate with *Jukurrpa* for flying termites and their pavements (*Jukurrpa* is described in Table 1; *Jukurrpa* is also known as Dreaming, but we use the former word). Many historical and recent artworks illustrate pavements, their regular spatial patterning in desert grasslands, flying termites and other features of termite ecology (Figs. 1b,d and 2d and Supplementary Figs. 1a, 4a–d and 6b).

Desert Aboriginal people, including Warlpiri and Anmatyerre (locations in Supplementary Fig. 3), conducted flying termite ceremonies to sustain and promote people–termite relations and productivity^12^. A ceremonial dance by Pitjantjatjara women, believed to increase production of a specific seed food, replicated the foot movements of threshing the seed in pits dug into termite pavements (Fig. 2c). As certain animal and plant-focused songlines and rituals span desert Australia, ceremonies that celebrated termites and their ecology were likely to be performed by other desert groups.

**Fig. 3:**
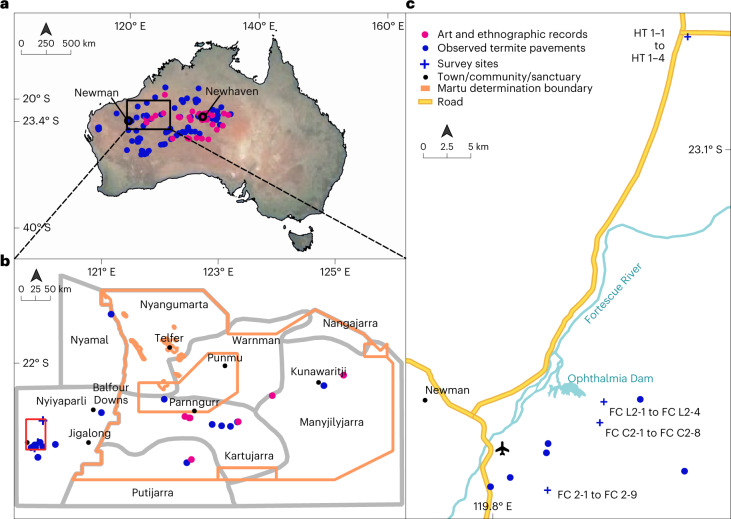
Art, ethnographic records and observations of termite pavements and winged termites overlap in distribution across arid Australia. **a**, Locations of Indigenous art and ethnographic records of termite pavements and winged termites and observed termite pavements across Aboriginal language groups in Western Australia, the Northern Territory and South Australia. Ethnographic records and pavement observations in New South Wales and Queensland have not been searched or mapped. **b**, Martu language areas and the Martu Native Title Determination Boundary (orange lines) with ethnographic records and pavement locations, with neighbouring Nyiyaparli lands to the west (boundaries approximate; shape files adapted from Kanyirninpa Jukurrpa). Language boundaries are in grey, and language names are shown in Supplementary Fig. 3. Note that all language boundaries are permeable, dynamic and often contested due to displacement, dispossession, relocation and other factors. Red boundary denotes map in **c**. **c**, Locations of plots surveyed in 2021 on Nyiyaparli country.

There are ethical and methodological challenges in interpreting Aboriginal art especially when the artists are deceased. As an example, paintings by Kaapa Tjampitjinpa (Fig. 1b), Michael Jagamara Nelson (Supplementary Fig. 4a, b) and other men were all titled ‘*Watanuma*’, as were paintings by the women Yuyuya Nampitjinpa and Wintjiya Napaltjari (Fig. 2d). We sought to understand whether they were all painting the same place or feature, and why women would be painting a men’s place and vice versa. From documentation, interviews and spatial information, we concluded that there are two sites known as *Watanuma*, located approximately 130 km apart, that are associated by the painters with different features of flying termites and termite pavements. *Watanuma* translates to both ‘edible flying termites’ and a ‘*Jukurrpa* site’. Satellite images show that both the *Watanuma* sites depicted by these paintings are surrounded by harvester termite pavements (mapped in Fig. 3). We look forward to speaking with living artists who continue to paint *Watanuma* and related subjects (for example, Fig. 1b,d).

Some Aboriginal artists also illustrated pavements co-existing with burn mosaics in grasses (Fig. 1a,c). The burning practices of Aboriginal people were critical to create and maintain diverse seral stages of spinifex grasslands^51^. Burning promotes a diversity of edible seed species that were then threshed on or in pavements (Ethnographic data, row 2; 10.26182/k3p0-hf57). Pavements in grasslands allowed desert women to unlock the energy and nutrients contained in the grasses that dominated their landscapes. Thus, the interactions between termite pavements, grasses, burning and food processing helped supply the carbohydrates needed to nourish the desert human populations, that expanded in the mid- to late Holocene^52^.

## The fairy circle debate

Scientists have debated causes of ‘fairy circle’ patterns in arid Namibia and Angola since the 1970s (refs. ^31,32^). In southern Africa, circles consist of bare patches, 2–24 m in diameter, within grasslands. Fairy circle origins have variously been attributed to eroded termite mounds, bacterial and fungal populations, toxic gases, *Euphorbia* toxins, insects (mostly termites^55^), plant self-organization or plant–termite interactions^32^. Very little of this literature explores the historical or contemporary knowledge of San, Khoi, Khoekoe-speaking or Bantu-speaking groups whose world view encompassed cultural landscapes that might explain fairy circles^56–58^. Initially, the circular bare patches attracted interest, then later the regular distribution of the patches was noted and measured; this patterning has attracted more attention in recent years^32^. Australian *linyji* on the land and in Aboriginal art also show regular patterning (Fig. 1).

From 2016 onwards, ‘fairy circles’ considered similar to southwest African ones were investigated in desert grasslands in north-western Australia^33,35,37^. Little termite activity was found, and those researchers saw no correlation between termite activity and the circles. The published works made no reference to Aboriginal knowledge about these phenomena and concluded that the spots were created by grasses self-organizing while competing for water and nutrients. These findings were amplified by Australian and international press^59,60^.

In contrast, Martu people are clear that the bare circles are occupied by termites, as also reported by Australian ecologists^41,42^. After alternative understandings of *linyji*^39^ were contested^38^, we collaborated further to do primary and secondary research into Martu knowledge of pavements and termites. We also examined artwork and published and unpublished information from other desert Aboriginal groups (Ethnographic data; 10.26182/k3p0-hf57). Regional and pan-continental distributions of the spot patterns and pavements were also mapped (Fig. 3a).

**Fig. 4:**
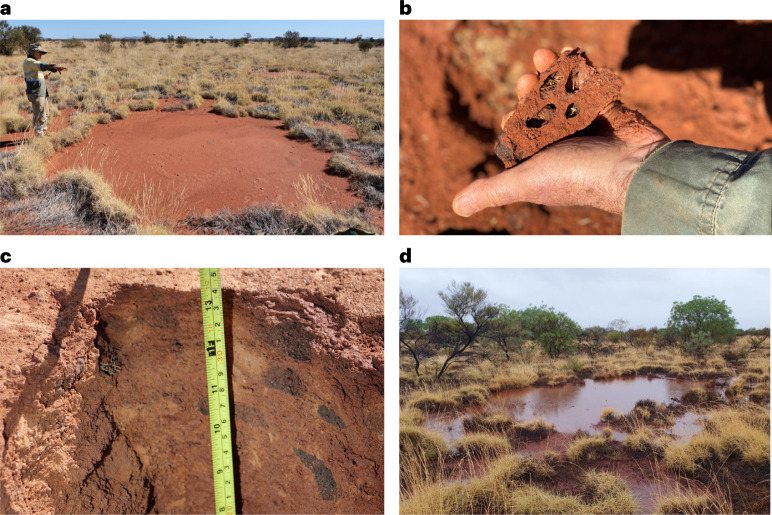
Aboriginal people’s knowledge was analysed in parallel to a survey of pavements on Nyiyaparli country near Newman. **a**, Typical pavement at FC 2 surveyed in same plot area as Getzin et al.^35^. This pavement averaged 5.2 m diameter with no mound. The area has dense spinifex grassland unburnt since at least 1985; such long-unburnt areas are now rare in arid Australia (photo © Fiona Walsh). **b**, Termite structure extracted from the pavement showing termite chambers, spinifex chaff stored in chambers, chambers with blackened walls, and all within dense consolidated soil (photo © Fiona Walsh). **c**, Dark termite frass-filled chambers and the dense termite cement distinguished the on-pavement structures to the inter-pavement soils that were loose and easy to dig. Frass chambers were 1–3 cm long (photo © Fiona Walsh). **d**, As foretold by Aboriginal people (Ethnographic data, row 15; 10.26182/k3p0-hf57), observations showed that termite pavements can hold rainwater at the surface for periods longer than the inter-pavement sandplain areas which absorb more quickly (photo ©Emma Stock; <6 km west of site FC 2-1). All images in this figure are covered by Creative Commons license CC BY-NC-ND.

In parallel to analysis of Aboriginal knowledge and art, we surveyed four of eight plots examined by previous researchers^35^ on Nyiyaparli country, east of Newman in the East Pilbara of Western Australia (Fig. 3b). Plots were distant from each other (Fig. 3b,c), but each contained multiple bare circles (for example, Fig. 1a). Across the four plots, we excavated 60 trenches down to 15 cm in 24 circles (for example, Fig. 4).

**Fig. 5:**
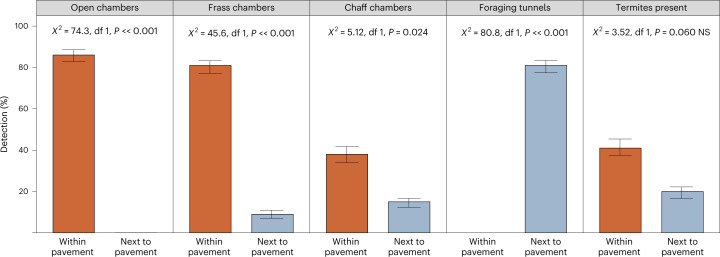
Termites and termite structures are much more common within and under pavements than in the spinifex grassland next to pavements. At the Nyiyaparli plots, 100% of trenches within pavements had open, frass and/or chaff chamber, and 41% had live harvester termites. Temporary foraging tunnels were found in the grassy inter-pavement areas. Termites and grass chaff were found in both, but at significantly different frequencies. Samples sizes: 23 pavements and 11 areas next to pavements (the former included 29 m of trenching in total). Error bars indicate standard errors. NS, not significant.

All 60 trenches (for example, Supplementary Fig. 5a–d) exhibited dense consolidated soils underneath a hard capping. On the pavement, the consolidated soil was dense and difficult to excavate by hand. By contrast, between pavements the spinifex sandplain soil was soft, friable and easily dug to 65 cm depth without any signs of consolidation. After air-blowing clean, 100% of trenches revealed termite chambers distributed horizontally and vertically; chambers were empty, some with black wall linings typical of termite plastering or filled with termite chaff (cut and stored grass), dark termite frass (filled latrines; Fig. 4b,c) and other materials. Galleries (passageways) connected chambers through the consolidated matrix. Termite foraging tunnels were found in the surface soils (0–5 cm) of the inter-pavement grasslands; these foraging tunnels originated and radiated from nearby pavements^61^. Foraging tunnels in the inter-pavement grassland areas were less consolidated, had no open chambers and had statistically significantly fewer frass-filled chambers (Fig. 5). We suggest that termites had cut and transported short sections of dry *Triodia basedowii* and other grass stems from the grassland areas, through the foraging tunnels and galleries, into chambers under the pavements^62^ (see Supplementary Information on termite ecology). Live grass harvester termites (*Drepanotermes perniger*) were found in 41% of the trenches (Fig. 5 and Supplementary Fig. 5e,f). We established unambiguous evidence that the fairy circles we surveyed were indeed *linyji* occupied by *manyjurr* (harvester termites), as Aboriginal people had said, demonstrated and illustrated.

We undertook further exploration of termite pavements on Ngalia Warlpiri country, at the Australian Wildlife Conservancy’s Newhaven Wildlife Sanctuary in the Northern Territory (Fig. 3a, Supplementary Fig. 6a–d). Here, pavements are called *mingkirri* or *parlarnji* in Warlpiri language.

Documentation from desert Aboriginal people reported and illustrated that pavements provide short-term sources of drinking water vital to them^63,64^ (Ethnographic data, row 15; 10.26182/k3p0-hf57). This cued our preliminary field tests. We found pavements had low discontinuous banks of sand buildup in spinifex hummocks at the pavement margins. The banks and consolidated pavement surface contribute to water ponding. As indicated by Aboriginal art and narratives, pavements functioned as ephemeral reservoirs of rainwater (for the same feature photographed in Nyiyaparli country near plot FC 2, see Fig. 4d). On the pavements, water pooled then infiltrated more slowly than in the adjacent sandplain. The dynamics of water on pavements is one aspect of ongoing research.

Having established an understanding of certain characteristics of *linyji*, including their consolidated subsurface texture and embedded chambers, we also observed eroded *linyji* in Mulga (*Acacia aneura, sensu lato*) shrublands. These shrublands were lower in the landscape than the relatively stable sandplain landform east of Newman airport (Supplementary Fig. 5g). The shrublands have developed along watercourses that are slowly eroding from sandplains that once contained intact *linyji*. This suggests that the Pilbara *linyji* may have great antiquity, possibly from the Pleistocene. Earthen termite nests in arid landscapes are known to persist for thousands of years after their inhabitants have died^65^ (see termite review in Supplementary Information).

Indigenous beliefs perceive human–ecological systems as tightly interconnected^26^. There may have been feedback loops between termite pavements, spinifex grasslands, Aboriginal burning practices, seed food production and responses of termites to those practices. It remains to be examined whether these could have co-evolved through ecologically transformative land-use practices^66,67^, as well as the condition and trend of harvester termite populations and their habitats under intensifying wildfire regimes and other threats in the Anthropocene or, as some Indigenous scholars prefer, the Capitalocene era^68^.

## Co-learning supports cultural resilience and ecosystem science

The principles and processes for collaboration, co-learning and co-research between scientists and First Peoples are well established^20,69,70^, though less well adhered to^30,71,72^. Our findings highlight that First Peoples’ knowledge can lead and inform, contrast and intertwine with scientific enquiry.

Conventional ecological studies have limitations in understanding slow processes in landscapes and ecosystems. In our case study about harvester termite pavements and their ecology, desert Aboriginal people’s deep-time knowledge and practices have added to a long-standing international debate about ‘fairy circles’, providing evidence contrary to a prevailing theory. Martu elders and other desert people revealed understanding, practices and stories about *linyji* circles in spinifex grasslands being occupied by harvester termites. Subsequently, when the scientists in our team excavated trenches on pavements, termite structures were always revealed. Thus, we found congruent lines of evidence to explain the ‘fairy circle’ phenomenon.

Aboriginal people’s ritual art and narratives also initiated new scientific questions about *linyji* (syn. *mingkirri*) and rainwater. Ethnographic evidence and subsequent field observations indicated that pavements hold water from rainfall until they overflow, evaporate or infiltrate. This water-holding capacity of pavements indicates an additional aquatic system for Australian desert people, plants, animals and ecosystems^40^.

Tarnita et al.^73^ hypothesized that termite–plant organization systems are necessary for regularly spaced bare circles to form. On the basis of our team’s findings, a broader range of human–ecological research questions can be explored relating to the relative magnitude of interactions between spinifex grasslands, harvester termites, soil, rainwater, anthropogenic burning regimes and edible grass seeds.

World-wide, First Peoples’ languages and detailed ecological knowledge are threatened by rapid ecosystem and economic changes^74^. Many First Peoples demand that younger generations learn about the cultures and Laws of their Country, and be supported to do so^75^. The urgency to strengthen traditional knowledge partly stems from its potential to support social and ecological resilience, and to help respond to current and future global changes^22,30^.

The co-development of partnerships between First Peoples and scientists can support the priorities of both. First Peoples benefit from participation and intergenerational transfer of traditional knowledge, while scientists benefit from information to test theories and catalyse new hypotheses to advance understanding for better environmental management. However, the challenges and compromises required for true co-learning between scientists and Indigenous people are substantial^76^. Scientists must recognize colonial impacts and improve on the inequities of neo-colonialism^5,6,68,70^. First Peoples and their knowledge are critical to improving ecosystem studies and ecosystem management; collaboration helps slow the decline of cultural and biological diversity on our planet.

## Methods

### Principles and ethics approvals

We are guided by cultural and research principles including of the Martu organization^77^, national and international ethical guidelines^78,79^, and Australian Indigenous people^69,80^. These principles include recognition, respect, self-reflection and cross-cultural capability, identifying personally with families, generosity, being serious and honest, substantive consultation or collaboration, translators as appropriate, accurate interpretation, supporting cultural integrity, informed consent, attribution, return and revitalization of findings, and maintaining indigenous culture. Future developments of our collaboration could include Aboriginal partnerships, benefit sharing, co-research, co-production and other practices.

Aboriginal co-authors of this paper are custodians of knowledge from their families and ancestors. They recognize the artworks, language names and uses of pavements described from other language groups as belonging to those groups. Other Aboriginal people also hold knowledge and custodial affiliations to animals discussed herein. Authorship here does not exclude rights of connection by others.

As in international contexts^70,81^, our approach is to bridge, weave and complement knowledge systems and recognize differences rather than extract Indigenous knowledge to subsume, integrate or commercialize it within a dominant system. Indigenous cultural and intellectual property (ICIP) has no specific protection laws in Australia, and hence there have been calls for stronger legal and moral rights^80^. An ICIP statement is provided at the end of this section.

The 1988–1990 ethnographic work among Martu (F.W.) was funded by The University of Western Australia, Australian Institute of Aboriginal and Torres Strait Islander Studies (AIATSIS) and the Aboriginal controlled organization, Western Desert Puntukurnuparna Aboriginal Corporation. Ethics permissions were provided by these organizations. Information from Martu was recorded and synthesized by Walsh (including 1990, 1992, 2009). It has been returned to Martu individuals and their representative organizations in various media and contributed to the successful Martu Native Title Determination^82^. Data in this paper derives from this past work and recent desk top research.

### Ethnographic methods

Aboriginal and non-Aboriginal team members used multiple methods, sources and media to gather and analyse Aboriginal People’s knowledge related to harvester termites and their pavements. This information was fragmented and widely dispersed. Communication with Martu and Warlpiri people and archaeologists, anthropologists, linguists, historians, art specialists and others, including recorded interviews, has yielded primary and secondary sources (Ethnographic data; 10.26182/k3p0-hf57). All Martu information recorded by F.W. is dated and traced to records from individuals or groups of individuals. We also searched Aboriginal language dictionaries, unpublished and published narratives, photo and film archives and other repositories (Ethnographic data; 10.26182/k3p0-hf57). Archival records and digital infrastructure hold substantive cultural information^83^. Tertiary sources in published ethnographies, biographies and more have been found. Primary to tertiary sources were compiled into a database of harvester termite-related content. Aboriginal artworks and associated documentation have been particularly informative. Similar methods are used in ethno-ecological research in Central Australia^84^. Triangulation and cross-checking corroborated, contrasted or clarified details. Further searches would yield more ethnographic information. We mapped the exact or approximate locations of ethnographic records to show the span of termite-related knowledge across language regions (Fig. 3a and Supplementary Fig. 2).

We have so far identified over 120 words describing flying termites, termite pavements and their environment, spanning 15 Aboriginal languages across arid Australia (Supplementary Fig. 2). These terms relate to termites and their human ecology, including specifically to harvester termites and pavements. Challenges in identifying records associated with harvester termites or their pavements include misidentifications and mistranscriptions by primary recorders of both termites and pavements and high synonymy in Western Desert languages. Ethnotaxonomy^85^ and further analysis and expansion of this vocabulary requires future work with language speakers and linguists.

Photographs and videos also provide evidence of Aboriginal uses of pavements. Links to three videos of Aboriginal women threshing on pavements are provided in Fig. 2. These are used with the permissions of key people in them, including Pantjiti McKenzie, Linda Rive and the Williams family of Parnngurr, and the organizations hosting those materials.

Desert Aboriginal artworks encode deep, rich, layered knowledge, some intertwined with ceremonies, sites and songlines^45,86^. Through peer-to-peer contacts, we interviewed, searched physical and online publications, and found example artworks and ethnographic records on the topic. The interpretation of desert Aboriginal art has challenges when artists are present and more so when they have passed away. The subjects and icons in paintings can be dynamic in context, space and time. Accurate documentation helps understand meanings that may be cryptic. To inform interpretations it is necessary to cross-reference to multiple works and artist lineages, art historians and language resources, geography and species ecology. Desert artists might repeatedly paint their same *Jukurrpa*. We compiled examples of some but not all of their works. We have not yet investigated flying termite-related icons in rock art^10^.

We cross-checked the artworks to our word list and ethnographic records. To determine the suite of uses and values attributed to harvester termites, these data were analysed and categorized thematically by uses, locations, people and artists associated with pavements, flying termites and more. Information locations are mapped in Fig. 3a. The ethnographic data table (10.26182/k3p0-hf57) synthesizes the uses of pavements and their termites, the number of citations found for each use and examples. Research into art to inform ecology is an evolving approach in desert Australia, and the methods will develop.

Aspects of Aboriginal knowledge about termites have prompted questions. Some knowledge was found to be commensurate with scientific information. Some remains ambiguous partly due to issues of translation; for example, do records of termite eggs being eaten refer to eggs or pre-emergent alates that have the same language name as eggs? There are issues of identification; for example, is this white ant likely to be a harvester or some other guild of termite? And, for example, does the 1980 painting documentation (Fig. 1d) ‘pupate stage of the flying [termite] *Watanuma*’ refer to soldiers and workers in spinifex or pre-emergent alates (that is, nymphs) in a pavement? Other Aboriginal knowledge elements have led to new lines of scientific enquiry; for example, why do desert people associate pavements with water sources? Some Aboriginal knowledge is incommensurate with scientific information; for example, *Jukurrpa* narratives about the shape and movement of termite ancestors in human form through landscapes. Not all knowledge and information from Aboriginal and scientific perspectives are convergent or assumed to be so.

The ethnographic research was compiled from many individuals who have since passed away. It provides a foundation for co-authors and potential relationships with the descendants of elders and scientists through knowledge comparison and co-research, cultural revitalization and artistic stimulation, land care and co-management. There are ethical complexities in comparing the knowledge of Aboriginal co-authors, their kin and ancestors to scientific knowledge; nonetheless, biophysical surveys were deemed necessary and thus were conducted.

### Biophysical survey, Nyiyaparli country

On 14–21 July 2021, we surveyed plots on Nyiyaparli country, east of Newman airport in the East Pilbara of Western Australia, near the Jigalong Road turnoff (Fig. 3c). Plot areas were selected for survey at four of the eight plots named and surveyed by Getzin et al.^33,35^. At plot FC L2, old survey markers indicated some circles had previously been examined by those researchers.

From our first pavement, subsequent pavements were selected by nearest neighbour proximity. We recorded latitude and longitude and the north–south and east–west diameter of each pavement. In total, we excavated trenches into 25 pavements in the surrounding spinifex grassland. Trenches were dug next to 11 pavements at 2 m distance from a pavement margin. In 16 pavements, three trenches were dug and in the remainder one trench was dug. All recorded trenches were 50 cm long, 15 cm wide and 15 cm deep. The first trench was dug in the centre of the pavement. Other trenches were on opposite sides of the centre trench halfway on each radius. In total, 29 m of trench was dug on the pavements.

We improved on previous methods^33,35^ by digging longer trenches to explore more of the pavement substructure, and using tools (mattock or crowbar, or 18 V electric power tool with an 8 cm shank blade) that were less likely to shatter the termite structures (compared with previously used jackhammers). After excavation, we used an 18 V air blower to remove excavation debris and dust to check for termite-related structures. In the future, alternative methods with heavier machinery are needed to examine the deep structure of the pavements. In the spinifex grassland next to 11 pavements, we cleared vegetation and then excavated three trenches dug at 50 cm long, 15 cm wide and 15 cm deep (as for pavements). We cleaned these with the air blower too.

Visual observations of the trenches included presence/absence of termite chambers, termite frass chambers, termite chaff and/or termite workers or soldiers. Each pavement and each trench was photographed. Qualitative observations were also recorded for each trench. At the end of surveying, all trenches were backfilled. The surfaces were flattened and raked flat, and spinifex returned over the sandplain trenches.

In terms of statistical analysis, each of five termite-related indicators (presence/absence of open chambers, frass chambers, chaff chambers, foraging tunnels and termite workers or soldiers) was tested for differences in probability of occurrence on pavements versus in areas next to pavements. A binomial generalized linear model was fitted, using the pavement or non-pavement area as the unit of replication, the numbers of trenches with and without the indicator on each pavement/non-pavement as the response, and site, pavement/non-pavement and their interaction as predictors.

### Biophysical survey, Ngalia Warlpiri country

Over 1–2 May 2021, we explored features of one pavement (approximately 1.5 m in diameter) on Ngalia Warlpiri country, at Australian Wildlife Conservancy’s Newhaven Wildlife Sanctuary in Northern Territory (Newhaven). One side of the pavement was vertically cut through to examine its internal structure and to collect termites. Two north–south trenches were dug into the pavement then connected by a tunnel under the full pavement (Supplementary Fig. 4c,d). The sandy soils surrounding the pavement were soft enough to dig by hand. The pavement was sprayed with 50 litres of water to observe water behaviour on the pavement.

### Termite species identification

Distribution maps indicated that both *D. perniger*^87^ and *D. rubriceps*^88^ occur near our sites in the east Pilbara and on Ngalia Warlpiri country, at the Australian Wildlife Conservancy’s Newhaven Wildlife Sanctuary (Newhaven). All termites from the Newman pavements were keyed and identified as *D. perniger*. Only one other species, *Schedorhinotermes derosus*, was found in one shallow chamber under spinifex adjacent to pavement FC2-7 near Newman. Termites were collected from the Newhaven excavations (Supplementary Fig. 4; note that the Newhaven sample size was small). *D. perniger* occupied both the Newhaven and the Newman pavements; however, pavements from these two localities showed very different structures (see Supplementary Information on termite ecology).

### Aerial methods

Google Earth images across the arid regions of Northern Territory, Western Australia and South Australia were used for broader-scale pavement observations and to detect pavement spatial patterns. We also gathered ground, helicopter and drone records provided by colleagues familiar with pavements. Satellite, aerial and ground observation locations are mapped in Fig. 3a. Further development of systematic methods for landscape scale aerial survey is required.

### ICIP statement

The artworks, photographs and videos in or linked to this paper remain in copyright to the artists and filmmakers; images are licensed under Creative Commons as indicated. The Aboriginal language terms and information used throughout the paper and the available datasets are attributed to relevant language groups and authors. Those publications identify co-authors and/or participants. These works and media were shared by Aboriginal people for learning and knowledge transmission to help teach their children and others, and to maintain their identity, languages, cultures and countries. We ask you to respect those intents and that, where citing, you attribute specific languages and sources as well as the authors of this paper. The information herein is not for commercialization or economic development without separate negotiations with relevant custodians and their representative agencies.

### Reporting summary

Further information on research design is available in the Nature Portfolio Reporting Summary linked to this article.

### Supplementary information

Supplementary Information Supplementary Figs. 1–6 and Text.

Reporting Summary

## Data Availability

The data used for Fig. 5 are available at 10.26182/k3p0-hf57. Two other datasets are available on the repository. These are a table of what Aboriginal and other people say, that is, ethnographic data sources and a wordlist of terms for termites in desert Aboriginal languages. Both are living documents, and the versions on the repository represent their status at the time of publication. Regarding the artworks and their documentation, we tabulated them and have permission to research but not to distribute them. Therefore, we cannot include digital copies of artworks in the data repository, but the art documentation is available on request.
