# Supplementary material for: First Peoples’ knowledge leads scientists to reveal ‘fairy circles’ and termite linyji are linked in Australia
Source: Nat Ecol Evol. 2023 Apr 3;7(4):610–22. doi: 10.1038/s41559-023-01994-1 (PMC10089917; doi:10.1038/s41559-023-01994-1)
Supplement: Supplementary file 1 — Supplementary Figs. 1–6 and Text. [file 41559_2023_1994_MOESM1_ESM.pdf]

nature ecology & evolution

Analysis

https://doi.org/10.1038/s41559-023-01994-1

First Peoples' knowledge leads scientists to reveal 'fairy circles' and termite *linyji* are linked in Australia

In the format provided by the authors and unedited

Supplementary Figures

a)

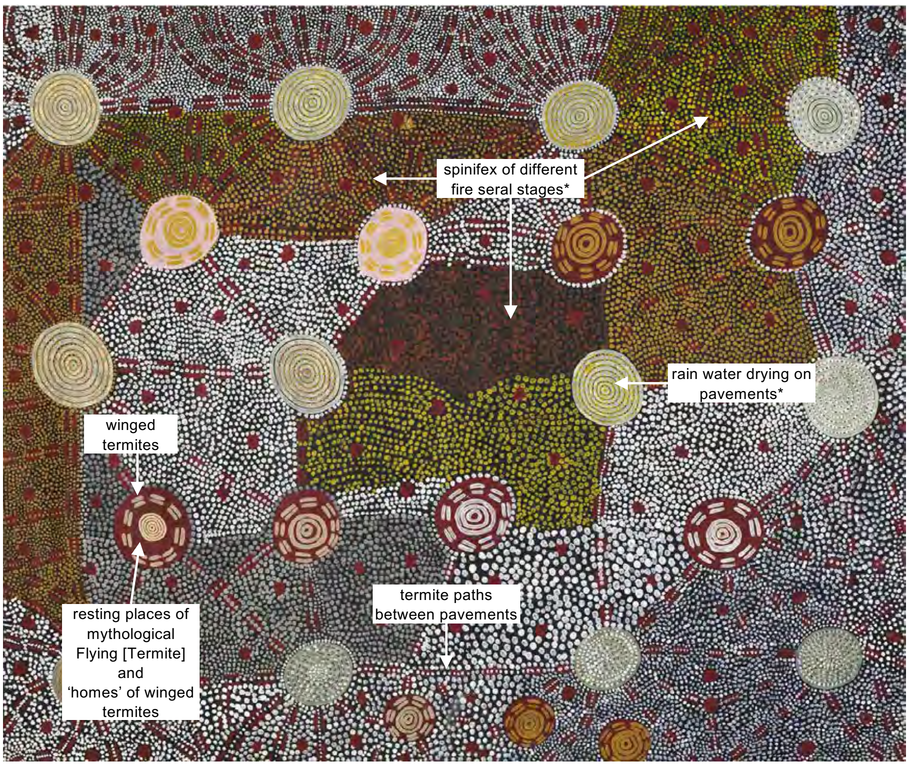

b)

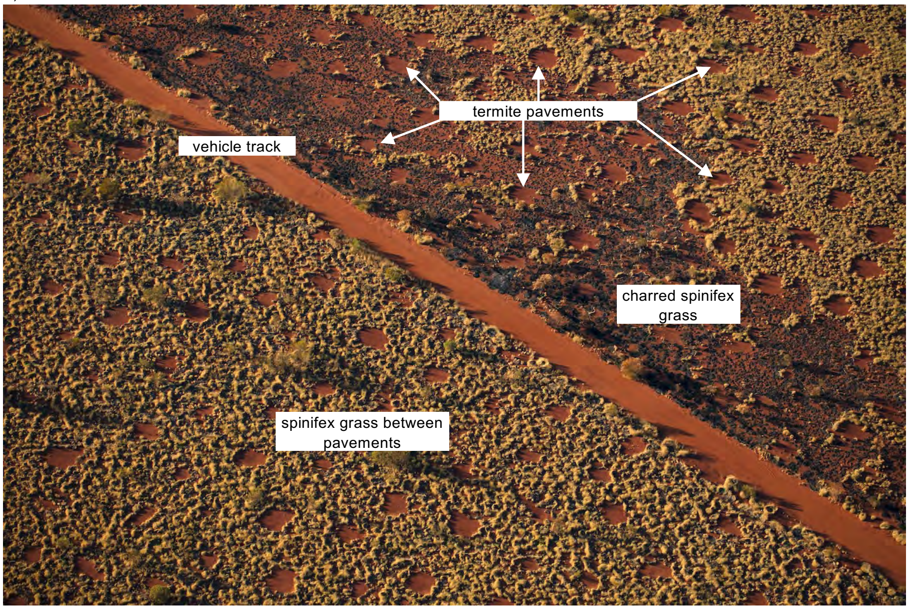

Figure S1. Further interpretation of some of the photos and paintings shown in Figure 1. a) Oblique aerial view of pavements amongst spinifex grassland. b) Icons in painting by Kaapa Tjampitjinpa (b. c. 1920, d. 1989). Whilst icons in Aboriginal art have multiple and context specific meanings there are some icons recorded when the paintings were documented, or icons that are commonly used[1]. An asterisk marks our interpretations derived from what Aboriginal people said about features of the pavements and threshing.

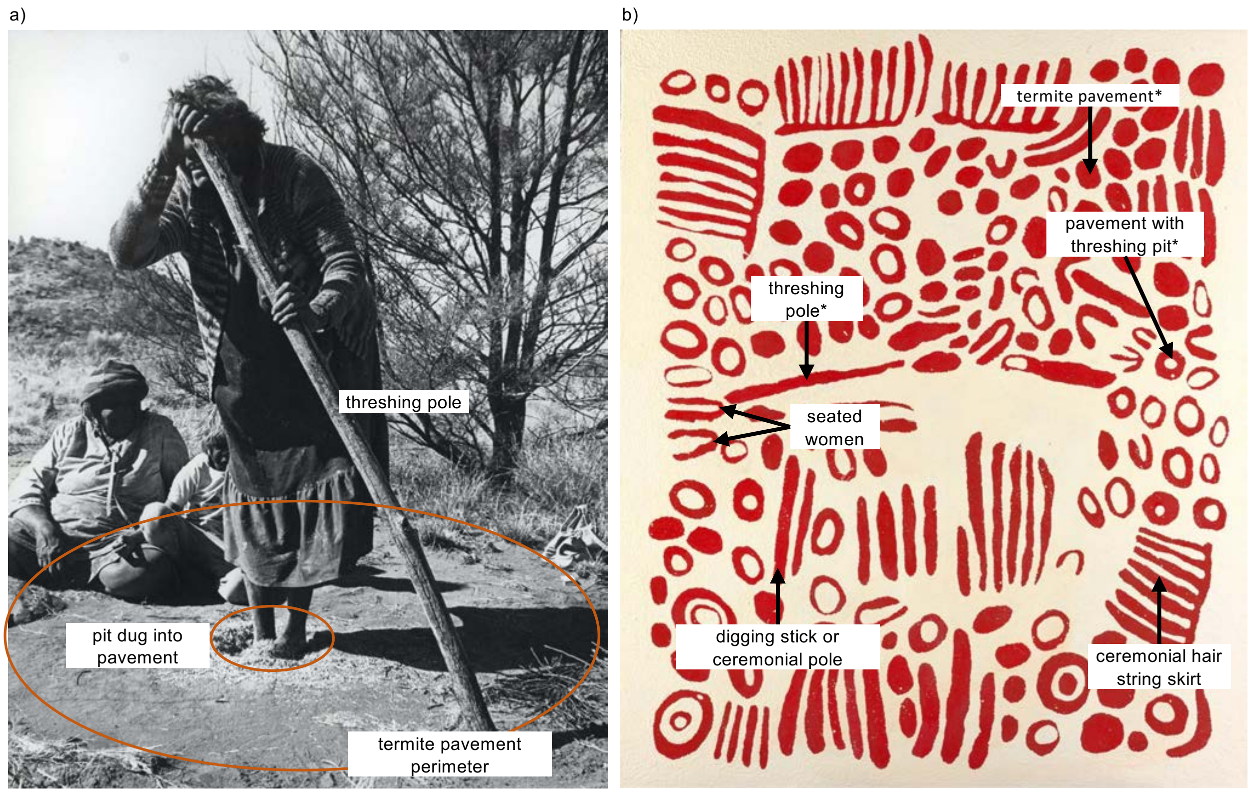

Figure S2. Further interpretation of some of the photos and paintings shown in Figure 2. An asterisk marks our interpretations derived from features of the pavements and threshing. a) Features of foot threshing of seed in a pit excavated into pavement surface. (photo by T. and B. Blake, 1987, ©Ara Iritija Archive Nos AI-0081679, see video Putu for Wangunu = Pavement for Seed Food, https://vimeo.com/654072562 and https://vimeo.com/539494391). b) Icons in painting by Wintjiya Napaltjarri (b. 1923 -1934, d. 2014). Icons in Aboriginal art have multiple and context-specific meanings; some icons are recorded when the paintings were documented and some icons are commonly used[1]. (© estate of the artist, licensed by Papunya Tula Artists and Aboriginal Artists Agency.) All images in this figure are covered by Creative Commons license CC BY-NC-ND.

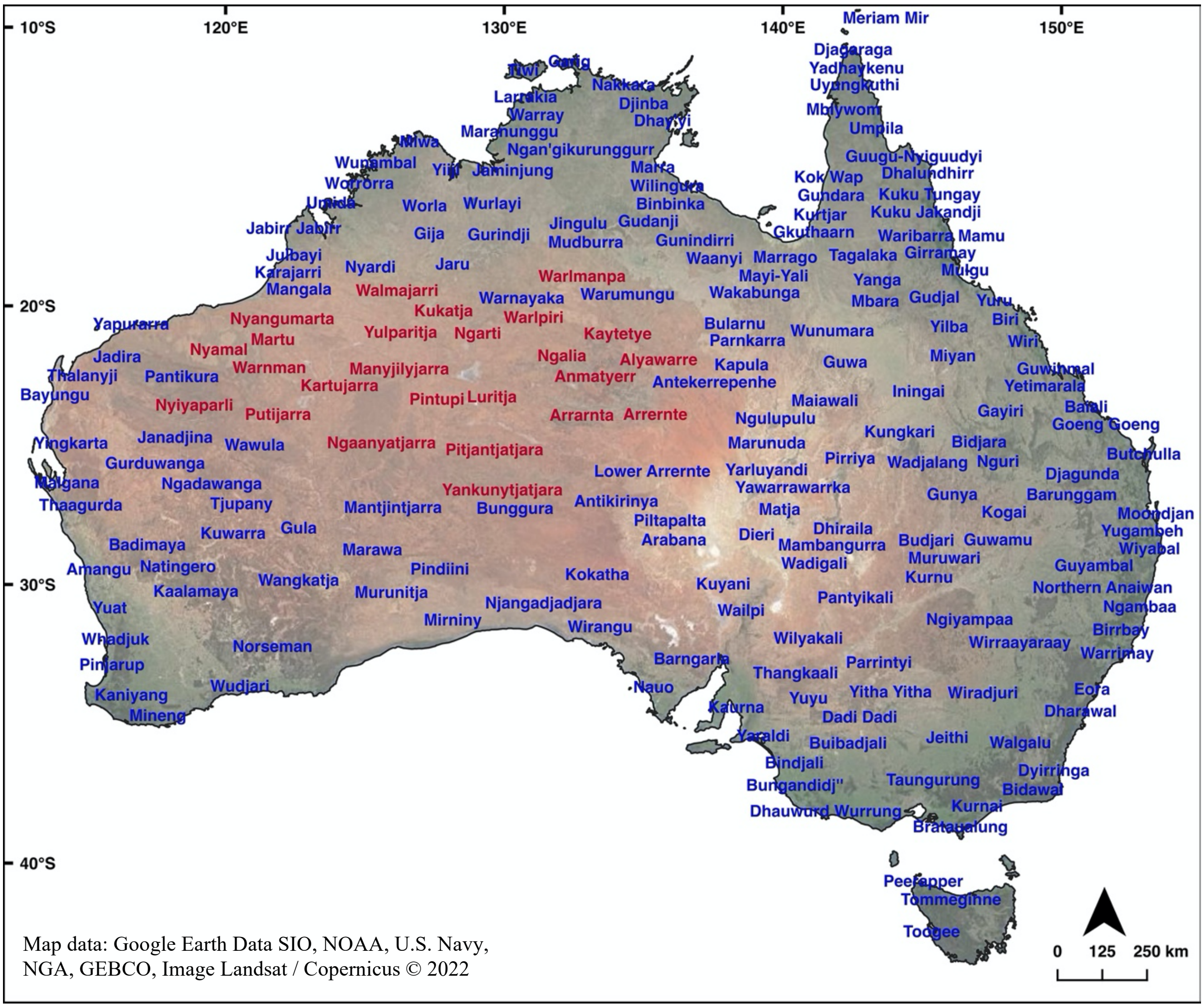

Figure S3. Indigenous languages of Australia. Languages referred to in the main article, supplementary section and extended data table in red. Data source: https://collection.aiatsis.gov.au/datasets/austlang/001. Data from Horton AIATSIS (1996) with changes made reducing the number of languages for clarity and to remove synonyms. All Indigenous language boundaries are permeable, dynamic. They are often contested due to the displacement, dispossession and relocation of Indigenous families and other factors.

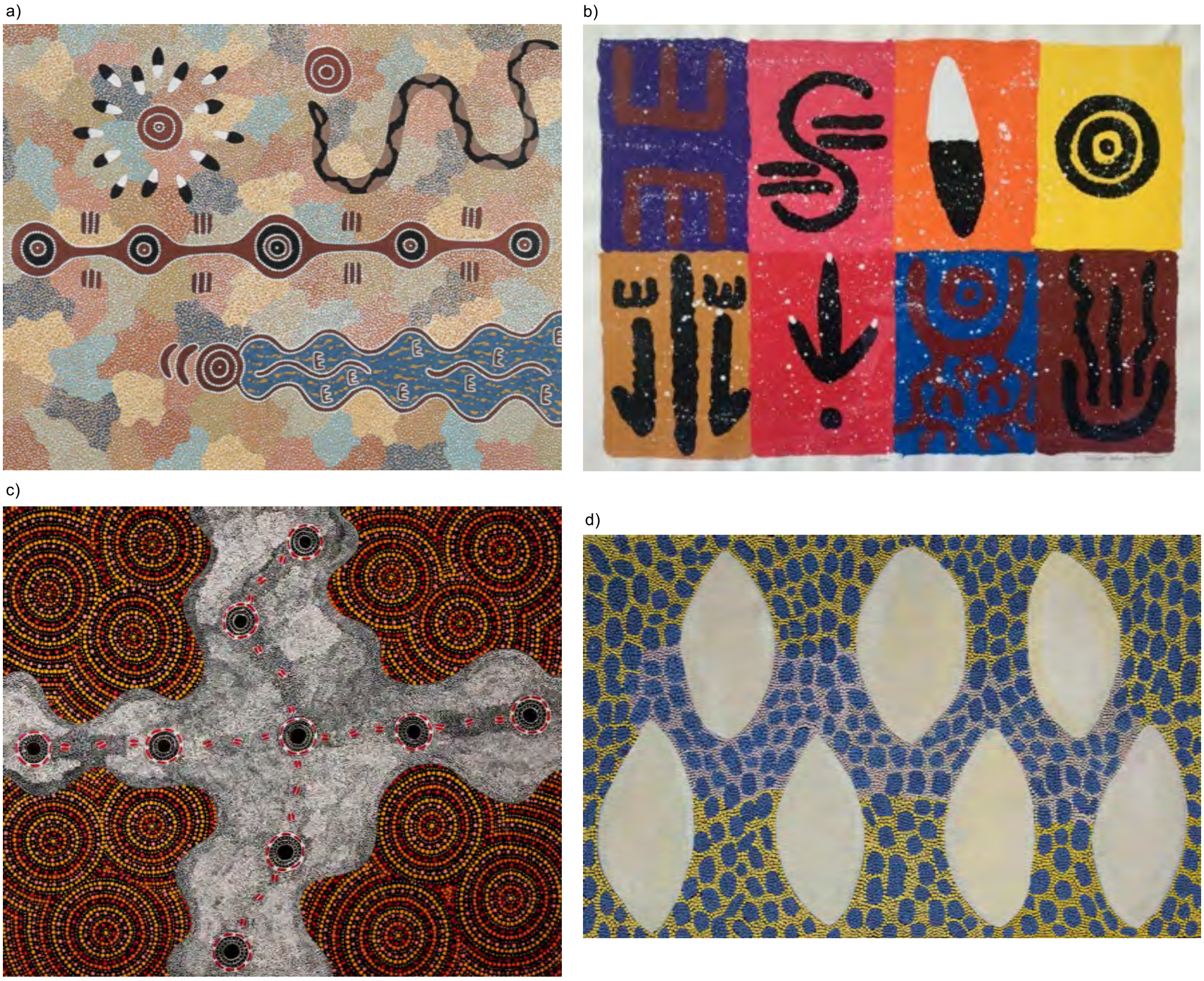

Figure S4 Examples of paintings by men and women and younger and older generations that portray termite pavements and associated features. a) Painting by Warlpiri Luritja man Michael Nelson Jagamara 1993 titled ‘Possum, Flying Ant [Termite], Stone Knives and Snake *Jukurrpa'* at Warntunguru north-east of Papunya. Acrylic on canvas, 152 x 122 cm. Collection of Morven Estate[2]. (© estate of the artist licensed by Papunya Tula Artists and Aboriginal Artists Agency.) b) Print by Warlpiri Luritja man Michael Nelson Jagamara 2001 titled ‘*Jukurrpa'*. Jagamara painted these *Jukurrpa* including the Flying Termite *Jukurrpa* throughout his career as a renowned Australian artist. The parallel bars in the top row are the Flying Termite *Jukurrpa*. Print on paper 14/20, 120 x 80 cm. (© estate of the artist, Oldham – Lavery collection.) c) Painting by Yuendumu woman Kara Napangardi Ross titled ‘*Pamapardu Jukurrpa* (Flying Ant [Termite] Dreaming) *Warntungurru'* 2022. Acrylic on canvas, 91 x 76 cm. (© the artist licensed by Warlukurlangu Artists.) d) Painting by younger Martu woman Bianca Simpson 2021, titled ‘*Kunurujanka'*. *Kunurujanka* has been recorded as one of three types of flying termites. Acrylic on canvas, 61 x 91 cm, 2021, (© the artist licensed by Martumili Artists.) All images in this figure are covered by Creative Commons license CC BY-NC-ND.

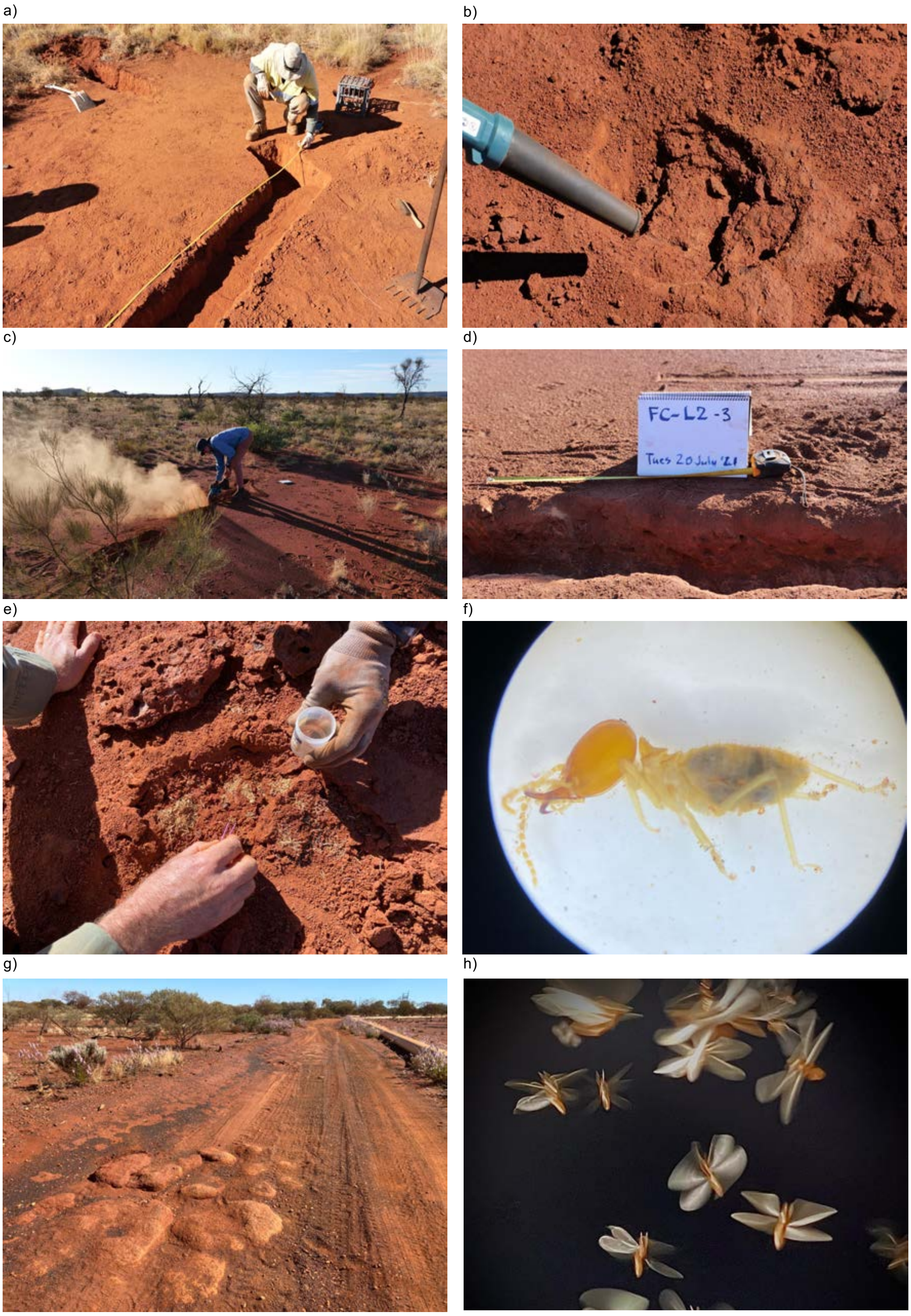

Figure S5. a) Large trench dug into pavement at plot FC 2 on Nyiyaparli country to test methods and observe subsurface structures. b) When the sand veneer was lightly blown away by an air blower, subtle features of the termitaria were revealed. c) Trenches were excavated and air blown, here at FC L2-2. d) Trench showing termite galleries and chaff amongst the consolidated matrices. e) Termite specimens were collected and identified. f) A collected soldier of *Drepanotermes perniger*. g) Consolidated domes indicate remnants of old pavements on eroding mulga shrublands exposed on tracks near the Fortescue River, Newman, W.A. (Photos a-g © Fiona Walsh). h) Flying termites or alates were gathered and eaten by Australian Indigenous people before and during swarming. Some artworks portray flying termites as double parallel bars indicating their wings. This is an unidentified species; photos or video of *Drepanotermes* alates are still to be sourced. (Photo © Josef Schofield).

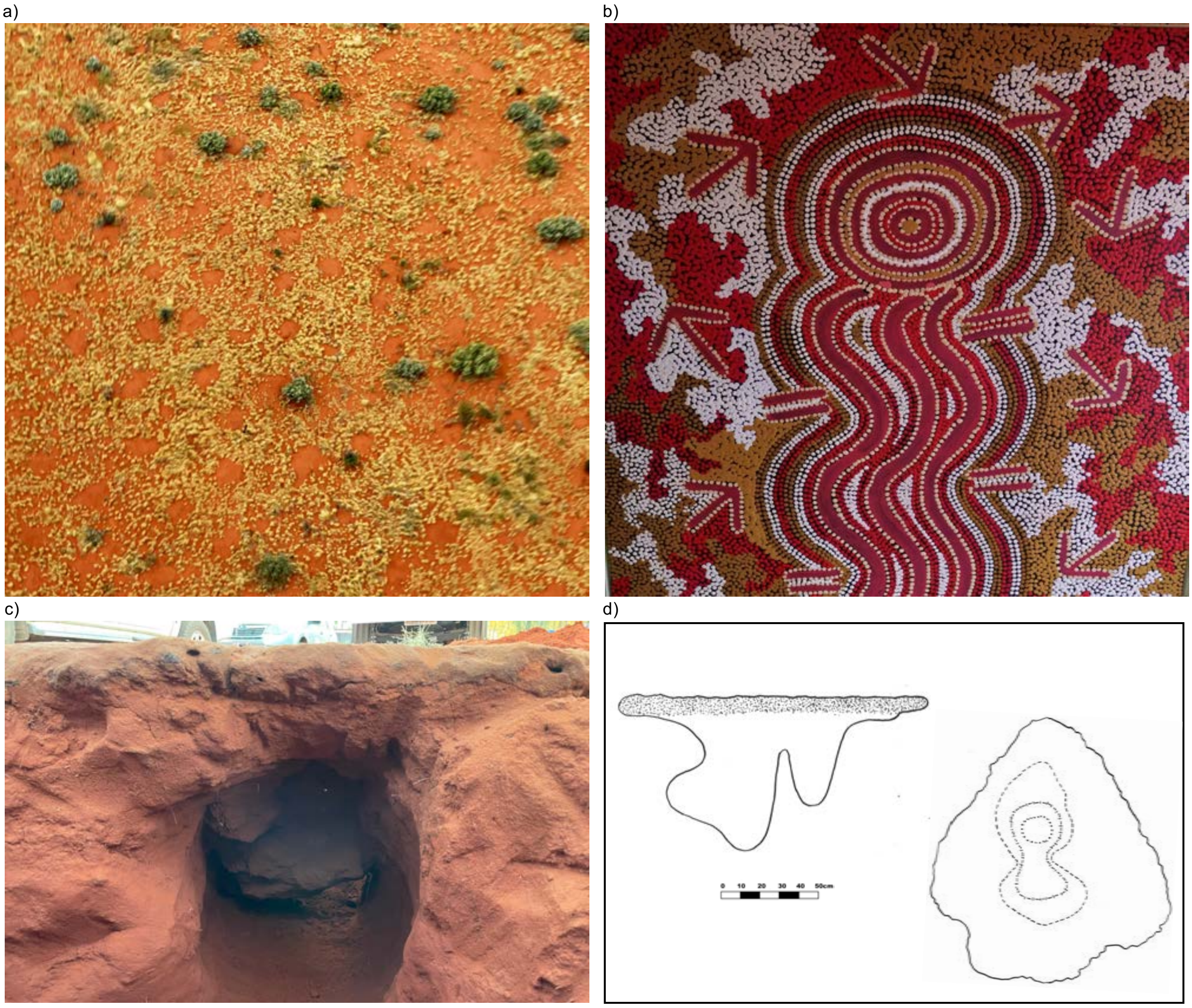

Figure S6. a) Pavements are common on spinifex sandplains of Newhaven. The first report nationally of termite pavements visible on Google Earth was by Australian Wildlife Conservancy in 2011. Pavements are seen on helicopter surveys for burning (photo © Josef Schofield). b) Section of artwork by co-author Lee Nangala Wayne shows flying termites and a co-relation with Emus and rain. Lee is also a Warlpiri Traditional Owner and works as a Ranger at Newhaven Wildlife Sanctuary. This artwork is Lees' Jukurrpa from her father's side. Her painting tells the story of rain (*ngapa*: wavy lines icon) filling waterholes (*karlampi*: concentric circles icon), which attracts Emu (*Yankirri* forked icon) and flying termites (*pamapardu*: parallel double bar icon). Acrylic on hardwood door 80 x 200 cm, 2019, (© the artist, Australian Wildlife Conservancy collection.) c) Tunnel dug under a termite pavement on Warlpiri Ngalia country, Newhaven, NT, sub-surface capping and subsurface consolidated soils (photo © Josef Schofield). d) Scale drawing of aerial and section views of pavement in Figure S4c by Sally Mumford. This pavement was 140 cm wide, 66 cm deep with a 7 cm thick capping. Pavement dimensions vary both within Newhaven and by comparison to Newman pavements. All images in this figure are covered by Creative Commons license CC BY-NC-ND.

Supplementary text on termite ecology and harvester termites from scientific publications

This section provides background scientific information about the ecology of termites and specifically harvester termites where relevant to our paper and the differing theories about the pavements, their formation and their spacing.

Nest types and occupants

All termites that live in soil build a nest, usually using a mixture of soils, termite faeces, and masticated and microbe-processed food (e.g., wood, leaf litter, grass leaves and stems). Most termite species (perhaps 90%) build their nests underground – so-called 'hypogaeic' or subterranean nests. Some species build their nests partially to fully above ground; these 'epigaeic' nests are usually referred to as 'mounds'[3]. Nests positioned just below the surface of the soil, so that the upper surface of the nest abuts the surface of the soil, are usually referred to as 'pavement mounds'. Their surface is flat, hard and resembles a pavement. Many species vary their nests depending on local conditions[4-7].

Pavement nests are found among the *Drepanotermes* grass harvester species in Australia[8-10]. They are not uniform, instead having various sizes, shapes and features. Some are flat; others have one or more small bumps or low mounds that may be positioned anywhere on the pavement. *Drepanotermes perniger* pavements range in size from 1- 4.5 metres[11]. Pavements may lie hidden under centimetres of wind-blown sand and thus be obscured.

One colony may build several pavement nests (i.e., they are polycalic) and castes move between them such that not all pavement nests in the set are occupied simultaneously. In north-eastern Australia, one colony of *Drepanotermes* was reported to have up to eight

nests[12]. Movement between pavement nests relates to harvesting of grasses around each nest. Foraging holes leading from underground tunnels to the soil surface are only maintained during periods of nest occupation. Sileshi & Arshad (2012) review and model the effects of foraging distance for harvester termites.

Mound ages and construction

Mounds may persist for long periods of time. For example, mounds built by *Macrotermes* spp in Africa were initially aged ca. 700 years old[13] but have since been aged to a maximum of ca. 2,200 years old. Mounds of *Microhodotermes*, a grass-harvesting species of arid areas in Africa, were aged to a minimum of 4,000 years[14]. *Syntermes* mounds in South America were aged from ca. 700 to ca. 3,800 years[15,16].

Australian mounds, including pavement mounds, have yet to be aged accurately. Termite mounds are energetically expensive to construct, given the building materials and size relative to workers and soldier castes thus mound longevity is advantageous. In Australia, one mound of *Nasutitermes triodiae* (the Cathedral mound termite) was observed to be at least 100 years old and was demonstrated to have had continuous occupancy of ca. 70 years[17]. When the Australian Overland Telegraph Line was constructed in 1872, the line passed over this mound. It was of substantial height and likely to have been decades old at the time. The top 1.8m of a mound was removed to allow passage of the lines. *N. triodiae* termites later partly rebuilt the top. When re-examined 98 years after the original damage caused by the telegraph construction, the mound was still standing (2.5 m high, though weathered). It had lost its *N. triodiae* colony but had gained two other termite species and one ant species[17]. Termite nests can be occupied by concurrent or successive species of termites[18,19] which indicates the origins of nests in deep time are difficult to determine. The longevity and

potential age of termite mounds means their origins are mostly deduced, rather than directly observed.

Spatial distributions of mounds

Termite mounds are typically evenly spaced when in uniform habitats[20]. Most studies have been conducted on large mounds in African or South American savannah. These have found termite mounds are evenly spaced; exceptions are due to non-uniform resources – or species lumping or misidentification[21-23]. In studies of mound distribution in Australia, including for *Drepanotermes*, mounds were regularly spaced (aka over-dispersed) in nine of ten sites[20] and six of twelve sites[24]. The larger and more uniform sites showed the greatest overdispersion, indicating that spatial scale and food resources are important determinants of pattern regularity[22].

Supplementary References

1 Johnson, V. Papunya Painting: Out of the Desert (National Museum of Australia Press, 2007).
2 Johnson, V. Michael Jagamara Nelson (Craftsman House, 1997).
3 Krishna, K., Grimaldi, D. A., Krishna, V. & Engel, M. S. Treatise on the Isoptera of the world: introduction. Bull. Am. Mus. Nat. 2013, 1-2704 doi:https://doi.org/10.1206/377.1 (2013).
4 Harris, W. V. Termite mound building. Insectes Soc. 3, 261-268 doi:10.1007/BF02224306 (1956).
5 Noirot, C. in Biology of Termites Vol. 2 (eds Krishna, K. & Weesner, F. M.) 73-126 (Academic Press, 1972).
6 Noirot, C. & Darlington, J. P. E. C. in Termites: Evolution, Sociality, Symbioses, Ecology (eds Abe, T., Bignell, D. E., & Higashi, M.) 121-139 (Springer Netherlands, 2000).
7 Grassé, P. P. Termitologia: Anatomie, Physiologie, Biologie, Systématique des Termites. Tome II. Foundation des Sociétés - Construction. (Mason, 1984).
8 Watson, J. A. L., Lendon, C. & Low, B. S. Termites in mulga lands. Trop. Grassl. 7, 121-126 (1973).
9 Watson, J. A. L. & McMahan, E. A. Polyethism in the Australian harvester termite Drepanotermes (Isoptera, Termitinae). Insectes Soc. 25, 53-62 doi:10.1007/BF02224485 (1978).
10 Watson, J. A. L. & Perry, D. H. The Australian harvester temites of the genus Drepanotermes (Isoptera: Termitinae). Aust. J. Zool. 29, 1-153 (1981).
11 Watson, J. A. L. & Perry, D. H. The Australian harvester temites of the genus Drepanotermes (Isoptera: Termitinae). Aust. J. Zool. 29, 1-153 (1981).
12 Holt, J. A. & Easey, J. F. Polycalic colonies of some mound building termites (Isoptera: Termitidae) in northeastern Australia. Insectes Soc. 32, 61-69 doi:10.1007/BF02233226 (1985).
13 Watson, J. P. A termite mound in an iron age burial ground in Rhodesia. J. Ecol. 55, 663-669 doi:10.2307/2258416 (1967).
14 Moore, J. M. & Picker, M. D. Heuweltjies (earth mounds) in the Clanwilliam district, Cape Province, South Africa: 4000-year-old termite nests. Oecol. 86, 424-432 doi:10.1007/BF00317612 (1991).
15 Martin, S. J., Funch, R. R., Hanson, P. R. & Yoo, E.-H. A vast 4,000-year-old spatial pattern of termite mounds. Curr. Biol. 28, R1292-R1293 doi:https://doi.org/10.1016/j.cub.2018.09.061 (2018).
16 Erens, H. et al. The age of large termite mounds—radiocarbon dating of Macrotermes falciger mounds of the Miombo woodland of Katanga, DR Congo. Palaeogeogr. Palaeoclimatolo. Palaeoecol. 435, 265-271 doi:https://doi.org/10.1016/j.palaeo.2015.06.017 (2015).
17 Watson, J. A. L. Old mound of the spinifex termite. Nasutitermes triodiae (Froggatt) (Isoptera: Termitidae). J. Aust. Entomol. Soc. 11, 79-80 (1972).
18 Kistner, D. H. in Biology of Termites (eds Krishna, K. & Weesner, F. M.) 525-557 (Academic Press, 1969).
19 Marins, A. et al. Termite cohabitation: the relative effect of biotic and abiotic factors on mound biodiversity. Ecol. Entomol. 41, 532-541 doi:https://doi.org/10.1111/een.12323 (2016).
20 Lee, K. E. & Wood, T. G. Termites and soils (Academic Press, 1971).

21 Darlington, J. P. E. C. The underground passages and storage pits used in foraging by a nest of the termite Macrotermes michaelseni in Kajiado, Kenya. J. Zool. 198, 237-247 doi:https://doi.org/10.1111/j.1469-7998.1982.tb02073.x (1982).
22 Davies, A. B. et al. Spatial variability and abiotic determinants of termite mounds throughout a savanna catchment. Ecogr. 37, 852-862 doi:https://doi.org/10.1111/ecog.00532 (2014).
23 Wildermuth, B. et al. Spatial patterns and life histories of Macrotermes michaelseni termite mounds reflect intraspecific competition: insights of a temporal comparison spanning 12 years. Ecogr. e06306 doi:https://doi.org/10.1111/ecog.06306 (2022).
24 Spain, A. V., Sinclair, D. F. & Diggle, P. J. Spatial distributions of the mounds of harvester and forager termites (Isoptera: Termitidae) at four locations in tropical North-Eastern Australia. Acta Oecol. 7, 335-352 (1986).
