# Supplementary material for: First Peoples’ knowledge leads scientists to reveal ‘fairy circles’ and termite linyji are linked in Australia
Source: Nat Ecol Evol. 2023 Apr 3;7(4):610–22. doi: 10.1038/s41559-023-01994-1 (PMC10089917; doi:10.1038/s41559-023-01994-1)
Supplement: Supplementary file 2 — Reporting Summary [file 41559_2023_1994_MOESM2_ESM.pdf]

nature portfolio

Corresponding author(s): Dr Fiona J Walsh

Last updated by author(s): 01/12/2022

Reporting Summary

Nature Portfolio wishes to improve the reproducibility of the work that we publish. This form provides structure for consistency and transparency in reporting. For further information on Nature Portfolio policies, see our Editorial Policies and the Editorial Policy Checklist.

Statistics

For all statistical analyses, confirm that the following items are present in the figure legend, table legend, main text, or Methods section.

n/a	Confirmed	
☐	☒	The exact sample size (*n*) for each experimental group/condition, given as a discrete number and unit of measurement
☐	☒	A statement on whether measurements were taken from distinct samples or whether the same sample was measured repeatedly
☐	☒	The statistical test(s) used AND whether they are one- or two-sided<br>*Only common tests should be described solely by name; describe more complex techniques in the Methods section.*
☒	☐	A description of all covariates tested
☒	☐	A description of any assumptions or corrections, such as tests of normality and adjustment for multiple comparisons
☒	☐	A full description of the statistical parameters including central tendency (e.g. means) or other basic estimates (e.g. regression coefficient) AND variation (e.g. standard deviation) or associated estimates of uncertainty (e.g. confidence intervals)
☒	☐	For null hypothesis testing, the test statistic (e.g. *F*, *t*, *r*) with confidence intervals, effect sizes, degrees of freedom and *P* value noted<br>*Give P values as exact values whenever suitable.*
☒	☐	For Bayesian analysis, information on the choice of priors and Markov chain Monte Carlo settings
☒	☐	For hierarchical and complex designs, identification of the appropriate level for tests and full reporting of outcomes
☒	☐	Estimates of effect sizes (e.g. Cohen's *d*, Pearson's *r*), indicating how they were calculated

Our web collection on statistics for biologists contains articles on many of the points above.

Software and code

Policy information about availability of computer code

Data collection	not applicable
Data analysis	Microsoft Excel version 16.61 spreadsheet for data storage and calculations of totals of recorded observations of sequentially numbered pavements and trenches within pavements and trenches next to pavement.<br><br>R statistics software version 4.1.3 for analysis by one-sided Chi-square test of above, as well as binomial standard errors of proportions / probabilities calculated as p(1-p)/sqrt(n).<br><br>Microsoft Excel version 16.61 spreadsheet for data storage of Aboriginal artworks, artists, dates and other parameters. We have permissions for publication of paintings in the paper only. We do not have permission to make artworks in full data set publicly available. We have only used the data set to indicate the numbers of paintings and numbers of artists found and stated that more are likely to be found. If essential that this data set be made publicly available then the 'artworks' column would be removed and only the text content remain.

For manuscripts utilizing custom algorithms or software that are central to the research but not yet described in published literature, software must be made available to editors and reviewers. We strongly encourage code deposition in a community repository (e.g. GitHub). See the Nature Portfolio guidelines for submitting code & software for further information.

Data

Policy information about availability of data

All manuscripts must include a data availability statement. This statement should provide the following information, where applicable:

- Accession codes, unique identifiers, or web links for publicly available datasets
- A description of any restrictions on data availability
- For clinical datasets or third party data, please ensure that the statement adheres to our policy

10.26182/e3xb-5h60 University of Western Australia data repository

Field-specific reporting

Please select the one below that is the best fit for your research. If you are not sure, read the appropriate sections before making your selection.

☐ Life sciences ☐ Behavioural & social sciences ☒ Ecological, evolutionary & environmental sciences

For a reference copy of the document with all sections, see nature.com/documents/nr-reporting-summary-flat.pdf

Ecological, evolutionary & environmental sciences study design

All studies must disclose on these points even when the disclosure is negative.

Study description	Our interdisciplinary and cross cultural methods had several strands. One synthesized 1) ethnographic records from Aboriginal people and knowledge holders about the use of termite pavements and/or termites 2) Aboriginal art works documented to be about termites and/or pavements.<br>This was compared and contrasted to the ecological strand of this study was a quantitative one to 1) expand the spatial area of pavements surveyed and reported in Walsh et al. 2016, and 2) to examine survey plot areas examined in the East Pilbara, Western Australia.
Research sample	In 2021, in arid spinifex grasslands we examined in situ and excavated trenches in bare circular areas (aka 'fairy circles' or 'linyji' and other Aboriginal names). We also compared trenches to soils next the bare areas. In 2016, we had concluded that similar features excavated in different locations were subterranean termite pavements or termitaria, as also recognised by Watson and Perry 1981 and others. Other researchers had rejected the regularly spaced bare circular features in the East Pilbara plot areas they surveyed as occupied by termites and argued for a plant self-organisation hypothesis as a cause.<br>The organism of our focus was harvester termites and their subterranean nests that have pavement surfaces and variable, low to no mounds. The harvester termites we collected were identified by termite specialist as Drepanotermes perniger.<br>We surveyed four plot areas within spinifex grasslands in the same locations as earlier researchers. Within each plot, we selected a bare circular area at the closest point to the latitude and longitude previously reported. From there we took a nearest neighbor method to other circles (aka pavements). We excavated both within each pavement and next to the same pavement in the inter-pavement area (Figure 4). We did not assume we would find evidence of termites. However, we used excavation methods that might reveal subterranean termite structures and features.<br>The sample size of four plot areas was determined so as enough data to be comparable to previous studies. The sample size was also influenced by the time, vehicles and personnel that were available over the 7 day period in the region.
Sampling strategy	A nested spatial hierarchy was surveyed with four plot areas then six pavements within each plot then three trenches within each pavement were excavated. Then a paired comparison was made of the three trenches within pavements and three trenches next to pavements.<br>We started with the pavement nearest the plot location recorded by previous researchers. We used a nearest-neighbour method to locate the next pavement and avoid bias. At the centre of each pavement, we recorded the pavement's latitude and longitude and marked and measured the north-south and east-west diameter of each pavement. In total, we excavated into 25 pavements. One of 25 pavements was a methodological pavement where we tested and refined our survey methods. In a pavement, the first of three trenches was dug in the centre of the pavement and the other trenches dug on the north-south centre line.<br>In the nearby spinifex grassland, the same dimensions of trenches were dug on a north-south line next to to 11 pavements two metres distant from the pavement.<br>In 16 pavements, three trenches were dug and in the remainder one trench was dug. The difference was due to reduction of field personnel from three people to one person and all trenches on pavements surveyed had evidence of termite structures before the loss of personnel. All trenches on pavements and adjacent to pavements were 50 cm long, 15 cm wide, 15 cm deep. In total, 29 m of trench were dug on the pavements, plus four metres in the trial pavement.<br>Trenches were dug using a mattock and crowbar and/or an 18 v electric power tool with an 8 cm shank blade. After excavation, each trench was cleaned with an 18V airblower on low speed to remove excavation debris and dust. This made soil and termite structures easier to see.
Data collection	In a pro forma field sheet records were kept of variables relevant to each pavement. One person (FW) recorded all the field data to promote consistency of recording. This was filled out in real-time by Fiona. Another researcher (PK) cleaned, observed and reported observations from all trenches for two field days. He reported the presence or absence of termite chambers, termite black inclusions (frass-filled chambers), termite chaff, foraging tunnels and/or termite castes. Along the length of the trench, he reported the soil density qualitatively (termite pavements: hard, solid and dense and so difficult to dig to the minimum 15 cm depth; vs inter-pavement areas were soft, loose and light and so easy to dig to > 50 cm depth), open chambers were roughly round, though often

irregularly shaped, diameter usually 10 – 20 mm), those chambers containing grass cut by the harvester termites (chaff) were recorded as were foraging tunnels (long, linear, roughly horizontal, diameter usually 5 – 8 mm), and the presence / absence of termites. Qualitative notes on the pavement, trenches and adjacent areas where also made. FW cleaned, observed and recorded observations for the other field days.
Every plot area, pavement and next to pavement was numbered and photographed with the date. All trenches were photographed. Each photograph is numbered back to the plot and pavement number. Real-time and 1" interval time-lapse videos with a camera on a tripod were made by FW concurrent to our survey on three pavements at three different plot locations.
Termites were collected and stored in ethanol in vials numbered to the plot-pavement. These termites were identified at The University of Western Australia by Theo Evans. Termites were photographed in the field and in the lab.
Primary field data was transcribed to Excel spreadsheet by FW. This was then aggregated. Statistical tests and other data summaries were done by co-author AS.

Timing and spatial scale

Field dates were July 14-21, 2021. This was in the cool season when there was more likelihood of harvester termites being near the surface, and milder conditions for field personnel to work safely. Three of us surveyed on three days then one of us surveyed on three days.
The field location is remote, being 1,180 km from Perth, the nearest city. Plot locations were chosen to coincide with the locations used by previous researchers as close as possible given hand-held GPS coordinates. On one occasion we inadvertently drove over a field peg probably left from a 2017 survey by previous researchers (the peg punctured our car tyre). This indicated the spatial accuracy of our site.
Pavement circles within a plot area were within a total area of less than one hectare. Plots were located several to tens of kilometres apart.
Intermittent ground, drone and Google Earth observations of pavements have been made at locations up to many hundreds of kilometers apart.

Data exclusions

No data were excluded from the analyses.

Reproducibility

If researchers revisited the same plots and pavements and re-applied our methods they would yield the same results.

Randomization

Nearest neighbour method to locate bare circles from the starting point determined by lat/long of previous researchers.

Blinding

It was not possible to blind field workers as sampling involved digging trenches in the soil either in the termite pavements in bare patches or from the inter-pavement areas. These areas are obviously different: bare soil vs grassy cover.

Did the study involve field work? ☒ Yes ☐ No

Field work, collection and transport

Field conditions

These lands have a mean average rainfall of 318 mm. Conditions during the study were mild and fine, clear skies and light winds. They were consistent over the study days e.g., at Newman WA on 19 July at 3pm it was 28C, 17% humidity, 15km/hr NE wind.

Location

In Western Australia, Pilbara region, Newman (see Figure 2 c) ; the plot area FC 2 was 6.47 km SE Newman airport runway. Pavement FC 2-1 was our methodological test pavement. Pavement FC-2-2 (our first experimental pavement) was at 119.856270000000 / -23.450300000000 then plot areas FC C2 9.45 km NNE of there with FC C2-1 at 119.910402800000 / -23.380621800000 then plot FC 2 was 2.4 km N at 119.914479042844 -23.359113257217 and the plot north near the Jigalong Road turnoff started at HT 1-1 120.000852224029 -22.983440481790.

Access & import/export

Early in 2021, Fiona Walsh made inquiries about land ownership and found the land area near Newman Airport was in transition from one entity to another. We sought permission from the prescribed body corporate on 17 June 2021. They verbally approved access. Regarding the collection of samples, we inquired with WA Department of Biodiversity, Conservation and Attractions and were advised that no permits were required.

Disturbance

We used a single off-road access track to and fro plot areas to reduce vegetation damage. Disturbance to termitaria, soils and vegetation were minimised. We used hand-held equipment only, not backhoes or other larger machines. We back-filled all excavated material into each trenches. We raked and leveled all disturbed soil. Grass hummocks were returned to their position. Flagging tape, markers and equipment were all removed.

Reporting for specific materials, systems and methods

We require information from authors about some types of materials, experimental systems and methods used in many studies. Here, indicate whether each material, system or method listed is relevant to your study. If you are not sure if a list item applies to your research, read the appropriate section before selecting a response.

Materials & experimental systems

n/a	Involved in the study
☒	☐ Antibodies
☒	☐ Eukaryotic cell lines
☒	☐ Palaeontology and archaeology
☐	☒ Animals and other organisms
☒	☐ Human research participants
☒	☐ Clinical data
☒	☐ Dual use research of concern

Methods

n/a	Involved in the study
☒	☐ ChIP-seq
☒	☐ Flow cytometry
☒	☐ MRI-based neuroimaging

Animals and other organisms

Policy information about studies involving animals; ARRIVE guidelines recommended for reporting animal research

Laboratory animals	Not applicable
Wild animals	Eight samples of 10 termites per vial were taken for identification by Prof. Theo Evans
Field-collected samples	Termite and termitaria samples are in secure, boxed and labelled storage at the University of Western Australia, School of Biological Sciences.
Ethics oversight	Samples are non-cephalopod invertebrates, and therefore animal ethics approval was not required for collection.

Note that full information on the approval of the study protocol must also be provided in the manuscript.
